# News and Notes

**Published:** 1898-09

**Authors:** 


### *NEWS AND NOTES.*

---

DR. VAN GOIDTSNOVEN has been elected Treasurer of the Atlanta Society of Medicine in the place of Dr. L. B. Grandy.

---

WE have received a most beautiful, as well as useful, half-year calendar from the Lactopeptine Company. Such "ads." always pay.

---

WE extend our sympathies to Dr. J. B. Baird, of this city, in the loss of his most estimable wife. She was a woman greatly beloved by a large circle of friends.

---

DR. HENRY BAK, a former resident of Atlanta but who for the last few years has lived in Chicago, has returned to the former city, and has again entered actively into the practice of his profession.

---

AN error occurred in a former issue of THE JOURNAL in stating that Dr. V. H. Taliaferro had moved to Eatonton, Ga. The doctor is located in elegant new quarters, 706, 708 English-American Building.

---

A MOST enthusiastic reunion of the alumni of the old Southern Medical College was held in Atlanta during the Confederate Veterans' Reunion. Good papers, followed by excellent discussions, were read by some of the members.

---

WE call attention to the article on Dr. Nicolas Senn, under the head of "Selections and Abstracts," in the last issue of THE JOURNAL. In it reference is fittingly made to our fellow townsman and eminent surgeon, Dr. J. McF. Gaston.

ON August 7, the Army Hospital at Ft. McPherson reported five hundred cases of typhoid fever. Patients have been so crowded and sanitary arrangements thus so hindered that a new camp, known as "Camp Hobson," has been established at Lithia Springs, Ga., so as to relieve the congested state at Ft. McPherson.

---

THE new Atlanta College of Physicians and Surgeons has become thoroughly organized and ready for the opening of the session in October. The new organization will occupy the buildings formerly known as the Atlanta Medical College, with, however, considerable additions which are now being added in the way of complete renovations. A large attendance is expected.

---

WE would most earnestly request that physicians all over the State send us items of news from their city or district. Deaths of physicians or marriages are often not known to the editor, and the same would be of interest to the profession all over the State. Notices of medical meetings will be cheerfully published, and thus we can find out what is going on in a medical way all over the State. Help us to make THE JOURNAL "newsy."

---

DR. LEONARD WOOD, whom many here in Atlanta remember when he was stationed at Fort McPherson as Assistant Surgeon, has been made military Governor of Santiago de Cuba. Dr. Wood, or General as is now his title, since distinguishing himself before Santiago at the head of the Rough Riders, was a great lover of athletic sports, and played for several seasons on the Technological football team. He was a fine specimen of physical manhood, and had many friends here in Atlanta.

---

WE have just received the new catalogue of the University and Bellevue Hospital Medical College. The Faculty consists of forty-seven Professors, Adjunct and Clinical Professors, besides Assistants and Instructors. We have not yet received the catalogue of the new Cornell University Medical School, but suppose that its Faculty will not be outdone in numbers. We suppose that such a Faculty must be organized on a purely philanthropic basis, and that is, to give all of its friends a showing in the new institution. Compensation cannot, of course, be thought of.

---

COL. RAY'S Regiment of Immunes has been found out to be a misnomer. This regiment was formed with the idea that the members who composed it would be immune to yellow fever, and therefore would be in a condition to stand the terrible fevers which were disabling many even of the Spanish soldiers in and around Santiago. As a matter of fact, very few of the members of the regiment were the "so-called immunes," but the whole body was made up of young men who perhaps had never had simple malarial fever. This was certainly true of the Atlanta delegation, and we understand it was almost true throughout the regiment.

---

THE Olmsted yellow-fever bill that we commented upon not long since in the *Bulletin* has at last been paid by the Atlanta Board of Health. It should never have been fought, as, under the circumstances, the amount was very reasonable. We congratulate Dr. Olmsted on receiving his check for $500 after so long a wait; and a vote of thanks is due the judge who stood by him when members of the medical profession were calling for a reduction. The reason doctor's fees average so much smaller than those of lawyers is that petty jealousy and spite seem to control the former more than the latter. A united profession would bring better pay and more respect to medical men than can ever be theirs while thus divided.—*Medico-Surgical Bulletin.*

---

THE United States Hospital at Fort McPherson has been crowded with soldiers from Santiago. There have been but few surgical cases, most of them being cases of fever. The "Ladies' Relief Association of Atlanta" has done much good by ministering to the wants of the wounded and sick. Some of the ladies have gone out every day to the Hospital, and in fact have almost acted as nurses. They are women who for the most part have never done such work before, but who have become so enthused that they nearly overdo the matter in their attention to the sick soldiers. A very amusing incident occurred a few days ago. One of the very intense enthusiasts went out, and on beholding the pallid face and weary look of one of the poor unfortunates, her sympathy immediately went out. "Can't I bring you some water?" she said; but the soldier shook his head. "Let me do something for you, let me bring you some milk or jelly"; and again the soldier shook his head. Finally, with one last effort, "Let me wash and bathe your face." With a weary look of pain the soldier murmured, "I will try; you are the seventeenth lady who has washed my face to-day." Enthusiastic women sometimes do too much.

---

PHYSICIAN wanting location address Lock Box 38, Adel, Ga.

---

DR. JNO. D. MARTIN, of Savannah, Ga., one of the oldest physicians in the State, died on August 3.

---

DR. R. T. HILLMAN, of Senoia, Ga., one of the most prominent physicians of the State, died of typhoid fever on August 6.

---

DR. E. R. ANTHONY, of Griffin, Ga., is spending a month or two in New York in attendance upon the numerous hospital clinics.

---

PERSONAL.—Dr. W. J. Green, of Clayton, Rabun county, Ga., will be in the State Senate November and December of this year. He wants a man of some experience to take his practice during that time. The field is good and pays well. Write to Dr. Green for particulars.

---

THE New Orleans Polyclinic will this fall have a "fall session"—an innovation in this school to which the attention of our readers is directed.

---

THE International Association of Railroad Surgeons recently held its annual meeting in Toronto, Canada, under the Presidency of Dr. Geo. Ross, of Richmond, Va. The next meeting will be held in Richmond, Va.

---

IN the death of Dr. Wm. Pepper, of Philadelphia, the profession at large has lost one of its most able exponents. Dr. Pepper was a man of world-wide reputation and the very backbone of the University of Pennsylvania.

---

THE daily papers report that cattle and horses which are pastured on Kings River, California, are suffering from Texas or splenetic fever. Within a few days 200 head of cattle succumbed to the disease, which is spreading rapidly.

---

CHARGES AGAINST A NEW YORK MEDICAL SCHOOL SET ASIDE.—The charges against the New York Post-Graduate Medical School and Hospital, relative to a misapplication of funds, have been investigated by the State Board of Charities and set aside as unfounded.

---

FOR URTICARIA.—Ord has found strophanthus, in five-drop doses, almost a specific in the chronic forms of urticaria. It is particularly indicated in the anemia of young women, especially if there is accompanying cardiac weakness with palpitation.—*Med. Times.*

TWO BILLS have been introduced in Congress, one providing for the appointment of Army Dentists, to have equal rank with surgeons; the other providing for the enlistment of one hundred female nurses to serve on board the ambulance ships and in the military hospitals during the continuance of the war.

---

GIFT OF A HOSPITAL TO THE RED CROSS.—Mr. James Armstrong, of New York, has offered the Red Cross Society the use of his country house at Centre Hill, Fla., as a hospital. The house has twenty large rooms, and is built on one of the highest points of land in the State, about seventy miles from Tampa.

---

DR. WILLIAM OSLER, Professor of Medicine at Johns Hopkins, is, according to the *N. Y. Medical Record*, "one of the fifteen scientists selected this year by the Committee of the Royal Society to be recommended for membership in the Royal Society of England." A very distinguished honor very worthily bestowed.

---

THE *Tri-State Medical Journal*, of St. Louis, reports the sudden death of a boy fifteen years old, which followed in thirty minutes an immunizing dose of antitoxin. The preparation was said to have been of the strength of 1,500 units. Between three and four cc. were used. The name of the manufacturer was not given.

---

IT is asserted by medical authority that there are more blind people in Spain, in proportion to population, than in any other country in Europe. At the beginning of the war it appeared that the whole nation was blind, but there is strong reason for the opinion that surgical operations by the United States will open their eyes. —*Medical Age*.

Announcement.—The partnership hitherto existing between Presley Blakiston and Kennith M. Blakiston, under the firm name of P. Blakiston, Son & Co., expired June 30, 1898, on account of the death of the senior member. The business of publishing, importing and dealing in medical and scientific books, as established in 1843, will be continued by Kenneth M. Blakiston, trading as P. Blakiston's Son & Co.

---

Sir John Lubbock has gone to the ant again, and if he keeps up his visits and others imitate him, that interesting insect will become useless for Sunday-school purposes. Sir John succeeded in getting fifty ants helplessly drunk and then placed them outside an ant hill. The sober ants came out, picked up their friends, and put them to bed to sleep off the effects of Sir John's liquor: the strangers, however, they sternly rolled over into the ditch.—*Scientific American.*

---

Yellow Fever Promptly Suppressed.—The Marine Hospital Service announced, July 9, 1898, that, as far as is known, there is not a single case of yellow fever in the United States. The total number of cases in the recent invasion at McHenry, Miss., was 24. The last patient was discharged July 8. There are no other cases under treatment and no suspicious ones under observation. Since this announcement was made we hear that cases of yellow fever have occurred among the troops in camp at Tampa.

---

A Special Committee Appointed to Commemorate Joseph O'Dwyer.—At the meeting of the Section on Diseases of Children, of the American Medical Association, held at Denver, Col., June 7–10, 1898, it was moved and carried unanimously that a Memorial Committee be appointed to commemorate the late Joseph

O'Dwyer, with suitable powers, etc., to collect such moneys and to act with other bodies for the same purpose. The committee is composed of the following: Dr. Louis Fischer, New York, Chairman; Dr. J. P. Crozier Griffith, Philadelphia; and Dr. F. E. Waxham, Denver, Col.

---

THE *Canadian Journal of Medicine and Surgery* has issued a special edition in its August number. It gives a very interesting and complete account of the meeting of the International Association of Railroad Surgeons, which was held in Toronto on July 6. Our Canadian reporter certainly wields a facile and entertaining pen, judging by the striking manner in which some of the occurrences are put. In speaking of that charming Southern gentleman, Dr. Geo. Ross, of Richmond, Va., the President of the Association, our Canadian reporter thus describes him: "A soldierly-looking, elderly man, with a white mustache and chin beard."

---

MOBILE and New Orleans have been, metaphorically, making faces at each other for some time over what is called "Louisiana's Inexcusable Quarantine." It is charged that Louisiana is quarantining places known not to be infected, and so violating every principle of true quarantine, and that, too, merely to satisfy the unreasonable fears of an ignorant public. The Mobile *Register* of July 8 says that "the light is breaking through the fog of the 'infernal quarantine' that afflicts this part of the world." Alabama seems to be converted to a national quarantine, and the papers are endeavoring to show that such a quarantine would not be opposed to States' rights. Will our States and cities adopt the Louisiana plan and keep out the brave boys from Santiago when they seek to return home because they have been exposed to yellow fever?—*Medico-Surgical Bulletin.*

THE European press has reported an operation that is probably destined to have much more than ordinary significance. The daughter of the Sultan of Turkey was operated on successfully for what would seem to have been hypertrophic stenosis of the pylorus. The operation was performed by Djemil Pasha, whom visitors to the surgical section of the recent International Medical Congress at Moscow will remember as an unassuming young man who, though the representative of the Turkish government, was only in evidence when he had something to say, which he did briefly and pointedly. After the operation he received the Osmanic order. Seven surgeons were present at the operation, and the dissemination of the knowledge of the operation among the Turks is likely to have a most salutary effect with regard to the medical and surgical treatment of Turkish women by men.—*Medical Age.*

---

A NOTICE.—It is contrary to the custom of the *Journal* to take its business into its reading pages, but the recent insolent effort of an agency of this city in the interests of a much-advertised proprietary preparation to demonstrate the venality of the American medical press cannot be permitted to go unnoticed. This journal's position requires no definition—the advertisement was unqualifiedly rejected. It was accepted, however, by a number of papers whose names have been widely circulated by this agency in violation of every tenet of decency in business dealings. The murder being out, and the impossibility of adequate explanation recognized, the names of the journals accepting the advertisement will be removed from our exchange-list. There is no other method by which disapproval can be so effectually demonstrated. We are pleased to note that the *Journal of the American Medical Association* has forced a retraction from the organ of this agency of the statement that it had accepted the proposition.—*Journal of Cutaneous and Genito-Urinary Diseases, July, 1898.*

